# Establishing Neuron-Specific Enolase Reference Intervals: A Comparative Analysis of Partitioned Approach- and Gender-Based Continuous Age- and Season-Related Models

**DOI:** 10.3390/diagnostics14192226

**Published:** 2024-10-05

**Authors:** Haibin Zhao, Dong Zhu, Miaomiao Zhang, Tengjiao Wang, Ning Han, Tinglei Ge, Xiaoming Ma, Anxin Wu, Runqing Li, Xiuying Zhao

**Affiliations:** 1 Laboratory Medicine Department, Beijing Tsinghua Changgung Hospital, School of Clinical Medicine, Tsinghua University, Beijing 102218, China; 2 Information Management Department, Beijing Tsinghua Changgung Hospital, School of Clinical Medicine, Tsinghua University, Beijing 102218, China; 3 School of Biological Sciences, University of Manchester, Manchester M13 9PL, UK

**Keywords:** neuron-specific enolase, continuous reference intervals, generalized additive models for location, scale and shape (GAMLSS), Hoffmann method

## Abstract

**Background/Objectives**: Static reference intervals (RIs) fail to capture the dynamic changes in bioanalytes. This study aimed to develop gender-based continuous age- and season-related RIs for neuron-specific enolase (NSE) using real-world data and to compare them with partitioned RIs. **Methods:** The NSE results from 4097 individuals were included after rigorous screening. Partitioned RIs were determined using the Hoffmann method. Generalized additive models for location, scale and shape (GAMLSS) were selected to develop continuous RIs. **Results:** The partitioned RIs are as follows: <16.4 µg/L for males aged ≥19 years; <14.47 µg/L for females aged 19–49 years; and <17.25 µg/L for females aged ≥50 years. For continuous RIs, NSE levels in males remain stable with age, while in females, NSE levels evidently increase around the age of 50. Although less impactful than age, seasonal changes still affect NSE levels. Dynamic changes and continuous RIs for NSE are visualized in this study. **Conclusions:** We developed gender-based continuous age- and season-integrated RIs for NSE in North China, highlighting the variation in NSE levels in females with age and season. Compared to static RIs, continuous RIs are more responsive to NSE, potentially enhancing the precision and individualization of health assessments.

## 1. Introduction

Cancer poses a significant threat to human health, with lung cancer still leading in incidence and mortality rates worldwide [1]. In particular, small cell lung cancer (SCLC) is highly malignant, with a 2-year survival rate of only 1% [2]. Neuron-specific enolase (NSE) is a useful biomarker for SCLC and is instrumental in diagnosing other neuroendocrine tumors that can occur outside the lungs, such as those in the pancreas, thyroid, thymus, and gastrointestinal tract [3,4,5,6,7]. Furthermore, NSE is employed in diagnosing a range of neurological diseases, such as cerebrovascular diseases, cranial injuries, ischemic brain diseases, and epilepsy [8,9].

Appropriate reference intervals (RIs) are crucial for ensuring proper diagnosis and treatment [10]. However, in China, there are currently no national standards or uniform guidelines for serum NSE, causing clinical laboratories to commonly rely on the RIs provided by manufacturers. Due to factors such as differences in ethnicity and geographic location, this practice carries a significant risk of being inapplicable in clinical application [11]. A previous study revealed that the RIs for NSE, established using data from a local population, differ significantly from manufacturer-provided IRs [12].

The traditional approach for establishing RIs, involving meticulously recruiting reference participants (the direct method), is often considered a “time-consuming, expensive and impractical endeavor” for most laboratories [13]. Consequently, data mining approaches (i.e., the indirect method) are increasingly being utilized to establish RIs [14]. The Clinical and Laboratory Standards Institute’s EP28-A3c document indicates that RIs can be established using the indirect method with stored laboratory data [11]. Partitioned methods are currently used to set RIs for biochemical analytes whose concentrations vary in terms of factors such as age and season. However, this static partitioning can oversimplify the dynamic and gradual changes, which could potentially result in inappropriate health assessment or even misdiagnosis. In response to this limitation, researchers have started studying continuous RI models for bioanalytes that vary with age [15,16,17]. However, to our knowledge, no studies have explored continuous RI models that incorporate multiple covariates, such as age and season (month), to adjust RIs for biomarkers.

This study aimed to examine the influence of gender, age, and season on NSE levels, develop gender-based continuous age- and season-integrated RIs for NSE using real-world data, and compare these RIs with partitioned RIs. Additionally, we explored the practical feasibility of implementing continuous RIs in clinical settings.

## 2. Materials and Methods

### 2.1. Study Subjects

**Figure 1:**
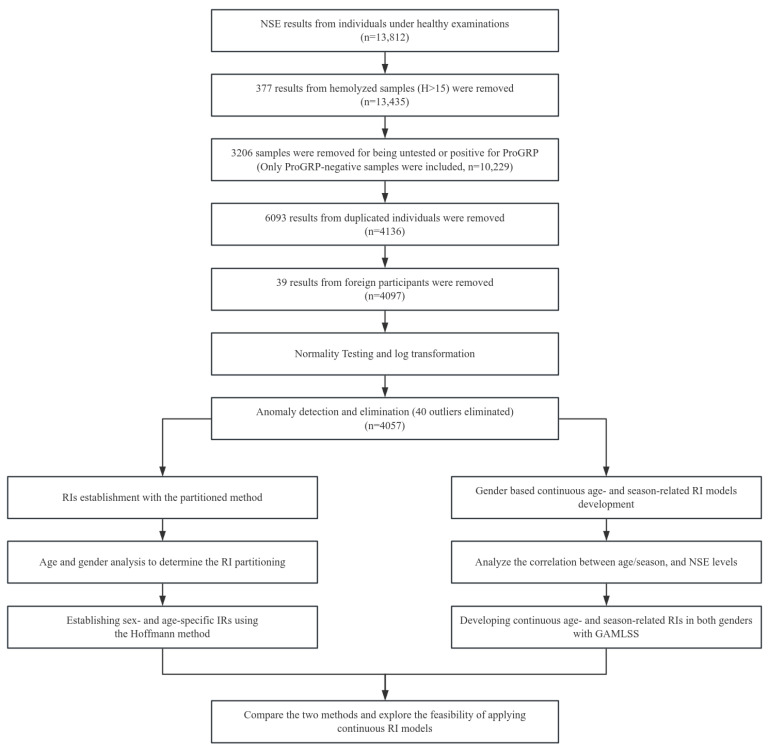
Screening procedures and general pathway of this study. Abbreviations: RIs, reference intervals; NSE, neuron-specific enolase; GAMLSS, generalized additive models for location, scale and shape; ProGRP, pro-gastrin-releasing peptide.

This study analyzed 13,812 serum NSE test results from individuals who underwent health examinations at Beijing Tsinghua Changgung Hospital between January 2020 and February 2024. Participants were required to be apparently healthy and have no disease records confirmed in the hospital information system at the time of blood collection. As hemolysis can lead to falsely elevated NSE levels, we only included samples with a serum hemolysis index below 15, indicating no hemolytic interference in the samples [18,19,20,21]. Additionally, as pro-gastrin-releasing peptide (ProGRP) offers similar diagnostic utility to NSE, only NSE data from individuals with ProGRP levels below 63 pg/mL, according to the manufacturer’s reference interval, were selected. Results from duplicate individuals and international participants were excluded. After rigorous screening, 4097 individuals—2270 males and 1827 females—aged 19 to 90 years were included. The screening procedures and study pathway are illustrated in Figure 1.

### 2.2. Detection of NSE, ProGRP, and Hemolysis Index

NSE was tested using the Cobas e801 immunoassay analyzer (Roche Diagnostics, Basel, Switzerland), along with original NSE test kits and calibrators. Quality control was performed using the Lyphochek Tumor Marker Plus (Bio-Rad, Hercules, CA, USA) at two concentration levels. ProGRP was tested using the Alinity i immunoassay analyzer (Abbott, Abbott Park, IL, USA), with original reagent kits, calibrators, and quality control materials, based on the chemiluminescence method. Quality control was performed at three concentration levels: low, medium, and high. Serum hemolysis index measurements were performed using the Cobas c702 clinical chemistry analyzer (Roche Diagnostics, Switzerland), along with original reagent kits, based on the spectrophotometry method. All tests were conducted according to the manufacturer’s instructions.

### 2.3. RI Partitioning

For partitioned RIs, the NSE results were first divided into two gender groups. Subsequently, each gender group was further divided into six age groups, resulting in a total of 12 groups (Table 1). The NSE results from all age groups were compared pairwise in males and females. In cases where no statistically significant differences were found, the corresponding groups were merged.

### 2.4. Statistical Analysis

#### 2.4.1. Data Preprocessing

The normality of our data was assessed using kurtosis and skewness measures. Data were considered normally distributed if the values for skewness and kurtosis were between −2 and +2 [22,23]. Log transformation was applied to skewed data where necessary to achieve a normal distribution. Outliers were identified and removed using the Z-score method, flagging data points with Z-scores beyond −3 or +3.

#### 2.4.2. Establishment of Partitioned RIs for NSE

We utilized the *t*-Test to compare NSE values between gender groups. The correlation between age and NSE results was assessed via Pearson correlation analysis [24]. The Tukey multiple comparison test was applied to conduct pairwise comparisons of NSE values among all age groups in both genders. The Hoffmann method was employed to establish discrete gender- and age-specific IRs for NSE [25,26]. One-sided RIs (upper limit only) were established due to the clinical significance. The significance level for statistical tests was set at 0.05.

#### 2.4.3. Development of Gender-Based Continuous Age- or Season-Dependent RI Models

The Tukey multiple comparison test was applied to conduct pairwise comparisons of NSE values among all month groups. The NSE values were plotted against age or month for both genders. The multivariate fractional polynomial regression method and generalized additive models for location, scale and shape (GAMLSS) were employed to capture the age- or month-dependent trends in NSE values. After comparative evaluation, the GAMLSS approach was selected for RI modeling due to its superior predictive performance, lower Akaike Information Criterion (AIC) and Bayesian Information Criterion (BIC) values, and significantly reduced edge effects. The degrees of freedom were automatically selected to ensure the model achieves a balance between smoothness and goodness of fit. We used cubic splines to smooth the relationship between age and NSE, while penalized splines were used to smooth the relationship between month and NSE. We used 10-fold cross-validation to determine the optimal smoothing parameter, and the models were fitted accordingly. The predicted 95th percentile line was estimated to represent the RI upper limit. These analyses were implemented using the “mfp” and “gamlss” packages in R (version 4.3.0).

#### 2.4.4. Development of Gender-Based Continuous Age- and Season-Integrated RI Models

To develop continuous RI models with dual-factor correction for age and season in different genders, we employed ordinary least squares regression, multivariate polynomial models, generalized additive models (GAMs), and the GAMLSS approach. These models were evaluated using the AIC, BIC, mean absolute error (MAE), and mean squared error (MSE), with lower values indicating better model fit. Residual Q-Q plots were used to verify whether the residuals followed a normal distribution in the GAMLSS (Supplementary Figure S1). The GAMLSS approach was ultimately selected for its superior performance. The degrees of freedom were automatically selected. Cubic splines were used to smooth the relationship between age and NSE, while penalized splines modeled the relationship between month and NSE. We used 10-fold cross-validation to determine the optimal smoothing parameter, and the models were fitted accordingly. Predictions of the upper 95th percentile for new data can be made by inputting age and month values into the fitted model. These analyses were implemented using the “stats” and “gamlss” packages in R. A 3D visualization of NSE trends and continuous RIs was created using the plot3D function in the “rgl” package of R (version 4.3.0).

#### 2.4.5. Software

All statistical analyses were conducted using R (version 4.3.0).

## 3. Results

### 3.1. Data Distribution and Anomaly Elimination

**Figure 2:**
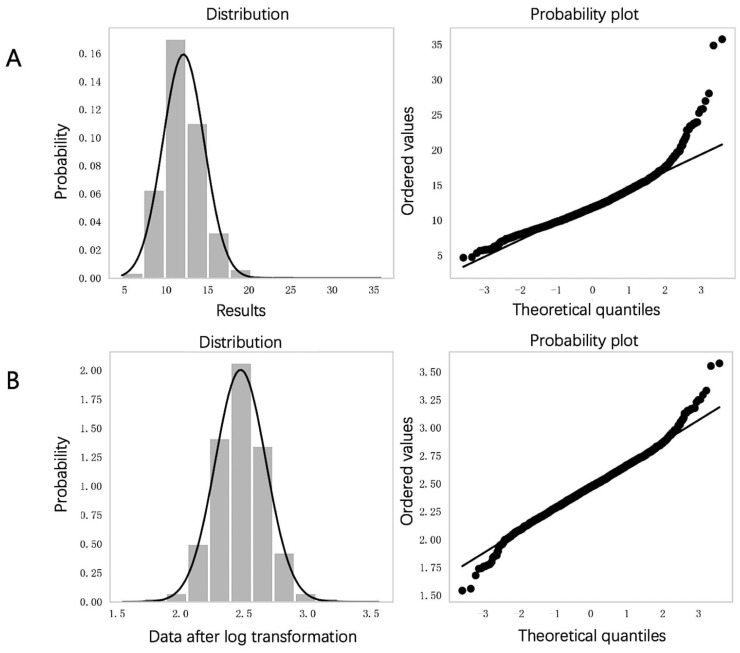
Distribution of NSE results. Part (**A**) illustrates the normality analysis of NSE results before transformation. Part (**B**) displays the normal distribution after log transformation. Abbreviations: NSE, neuron-specific enolase.

After screening, NSE results from 4097 individuals were considered. Initial data exhibited skewness (skewness 1.265; kurtosis 5.969; Figure 2A). Log transformation normalized the distribution (skewness 0.105; kurtosis 1.22; Figure 2B). We identified and removed 40 outliers, resulting in a total of 4057 cases from 2243 males and 1814 females aged 19 to 90 years.

### 3.2. Gender and Age Grouping Analysis

Significant statistical differences in NSE values were observed between gender groups (*p* < 0.001), necessitating gender-specific RIs. Pearson correlation analysis revealed no significant correlation between NSE levels and age in males (r = −0.052), whereas a moderate positive correlation was found in females (r = 0.325). Multiple comparison tests confirmed these findings, showing no significant differences in NSE levels among male age groups, whereas significant differences were found among female age groups, necessitating age-specific RIs for NSE (Table 2). Thus, males were consolidated into one group, while females were divided into two age-based groups: one comprising individuals aged 19–49 years, and the other comprising individuals aged over 50 years.

### 3.3. Sex- and Age-Specific RIs with the Partitioned Approach

The sex- and age-specific RIs for NSE, determined via the Hoffmann method, are as follows: <16.40 µg/L for males ≥ 19 years; <14.47 µg/L for females 19–49 years; and <17.25 µg/L for females ≥ 50 years.

### 3.4. Gender-Based Continuous Age-Dependent RIs

**Figure 3:**
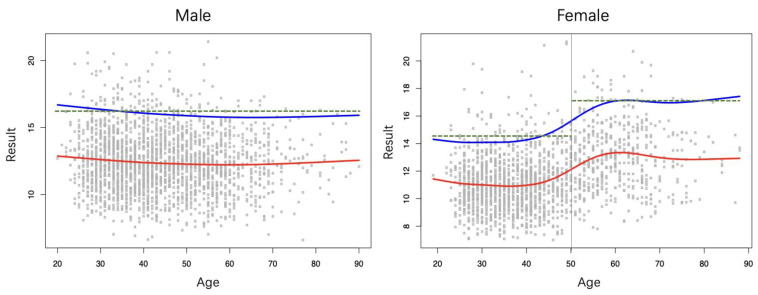
Continuous age-related RI models for NSE. The red line is the fitted curve of NSE values to illustrate the NSE trend with age. The blue line is the predicted 95th percentile fitted curve, representing the upper limit of NSE RIs. The dashed green lines represent the upper limits of partitioned RIs we established.

The age-related RI models developed with GAMLSS are visualized in Figure 3. In males, NSE levels are stable, with the RI upper limit peaking at 16.69 µg/L at 20 years and the lowest value being at 15.75 µg/L at 66 years, close to the overall RI of 16.40 µg/L established via the Hoffmann method. In females, NSE levels increase with age, particularly around the age of 50. The RI upper limit trend shows a nadir of 14.08 µg/L at 27 years and a sharp rise from 40 to 60 years, peaking at 17.14 µg/L at 63 years and stabilizing at high levels.

### 3.5. Gender-Based Continuous Season-Dependent RIs

**Figure 4:**
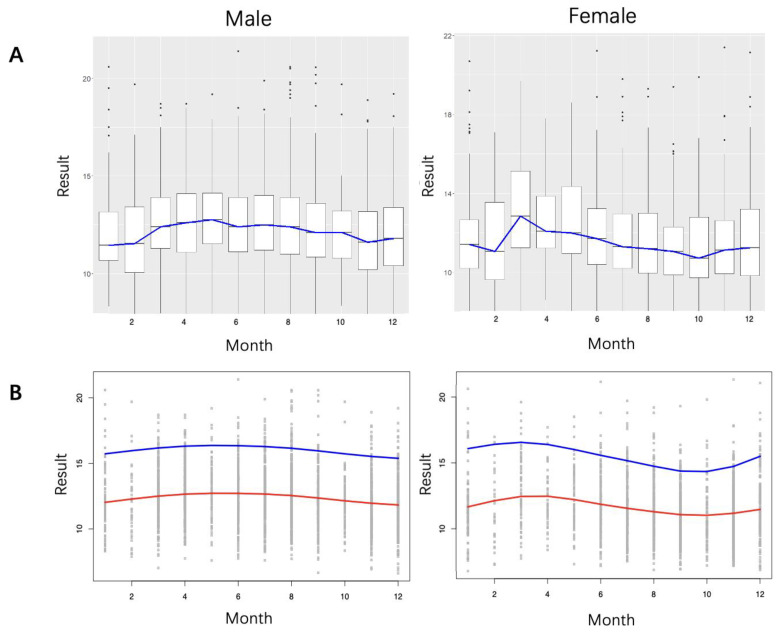
Continuous season-related RIs for NSE. (**A**) Boxplots were used to display the distribution of NSE results for each month, with a blue line connecting the medians of NSE in each month to illustrate the trend. (**B**) The red line is the fitted line to illustrate the trend in NSE values with month. The blue line of the predicted 95th percentile represents the upper limit of the NSE RIs.

The multiple comparison test revealed significant differences among month groups in both genders (Supplementary Table S1). The boxplots illustrated the seasonal trend regarding monthly NSE value distributions (Figure 4A). GAMLSS modeled the relationship between NSE value and month (Figure 4B). Although less impactful than age, seasonal changes affect NSE levels, with higher values appearing in spring and lower values appearing in autumn and winter. This fluctuation is more pronounced in females, with the RI upper limit peaking in March at 16.69 µg/L and reaching its lowest value in October at 14.49 µg/L. Males show a relatively stable pattern, peaking at 16.38 µg/L in May and falling to 15.40 µg/L in December.

### 3.6. Gender-Based Continuous Age- and Season-Integrated RIs

**Figure 5:**
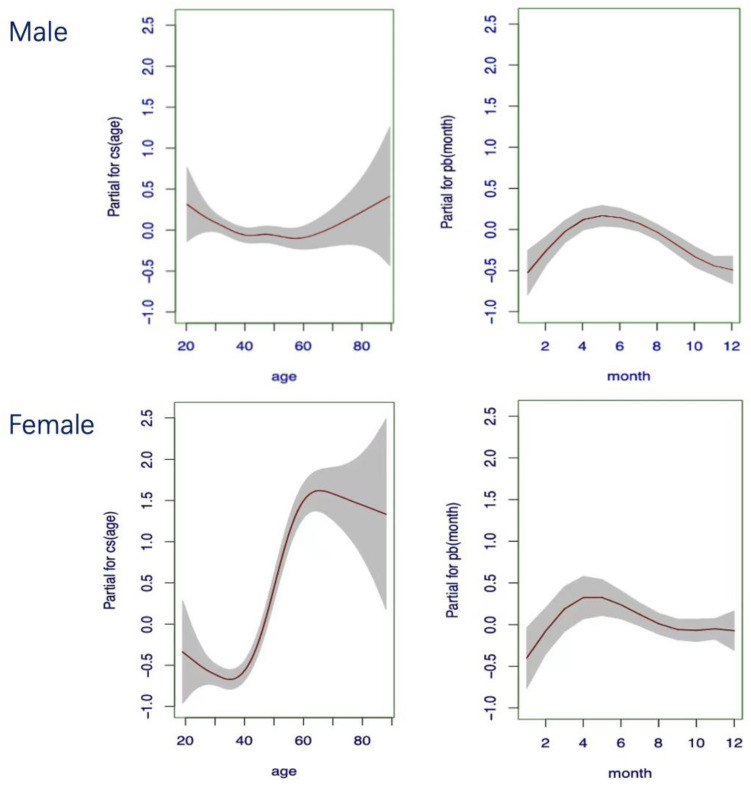
GAMLSS for gender-based continuous age- and season-integrated RIs. The term plot represents the estimated effect of a predictor variable (age/month) on the response variable (NSE values). The Y-axis depicts the estimated effect of a predictor variable on the response variable, encapsulated by the smooth function (cubic splines or p-splines) for that predictor. The X-axis shows the value of the predictor variable. The upper 95th percentile representing the upper limit of NSE RIs can be derived using the GAMLSS approach by inputting age and month values.

**Figure 6:**
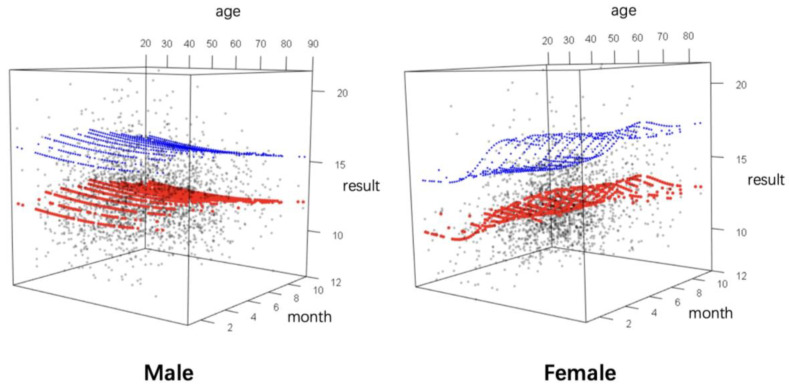
Three-dimensional visualization of NSE trends and IRs by age and month. The three axes of the cubes represent the age, month, and NSE values, respectively. The NSE results from the original dataset are represented in black dots. The red dots indicate the fitted NSE values at different ages and months. The blue dots indicate the fitted 95th percentiles, representing the upper limit of RIs. These dots form comprehensive surfaces that reflect the NSE trends by age and month in different genders.

Considering multiple influencing factors, we developed NSE continuous RI models with dual-factor correction for age and season in different gender groups using GAMLSS. Term plots were employed to visualize the non-linear relationships captured by GAMLSS, demonstrating how each predictor variable influences the response (Figure 5). The trends observed in the dual continuous variable-corrected RI models were consistent with those using single-factor correction. In summary, males are less affected by age and month, whereas females show more pronounced influences, primarily derived from age. NSE levels increase with age, particularly around the age of 50. By inputting age and month values into the fitted model, we can predict the gender-, age-, and season-specific RIs for NSE. To provide an intuitive understanding of these trends, we created a 3D visualization, shown in Figure 6. In this visualization, the fitted NSE values from the binary GAMLSS-based model are plotted in red, and the upper limits of the RIs are marked in blue (Figure 6).

## 4. Discussion

In this study, we established partitioned RIs specific to sex and age for NSE. Our hospital, located in China’s capital, collected data from a diverse North China-based demographic, making our RIs broadly applicable across North China. This is, to the best of our knowledge, the first study to establish the RIs of serum NSE in a large sample size in North China. Given that geographical variations typically have minimal impact on oncological biomarkers, these RIs have the potential for application in laboratories across other regions. However, considering that ethnicity is also a potential influencing factor, caution is warranted when applying these RIs in regions with significant ethnic minority populations. According to Clinical and Laboratory Standards Institute’s EP28-A3c document, it is important to ensure systematic comparability and RI validation prior to RI adoption, especially in such regions. For detailed protocols on RI validation, please refer to the EP28-A3c document [11].

Furthermore, we developed gender-based continuous age- and season-integrated RIs in this study. To the best of our knowledge, this is the first study to develop continuous RI models that simultaneously incorporate multiple continuous covariates, specifically age and month, for biomarkers. This approach accounts for both age and month in capturing the continuous effects on biomarkers and better reflects the combined influence of multiple factors on RIs, rather than considering age alone, exemplifying the core tenets of precision medicine.

The reliability of RIs established using real-world data depends on careful data assessment and the application of robust statistical methods to eliminate “data noise”. To ensure accuracy in NSE measurements, only samples with a serum hemolysis index below 15 were included [18,19,20,21]. ProGRP, as a biomarker closely associated with neuroendocrine tumors, shares similar diagnostic utility with NSE [27,28,29]. Thus, we only included data from individuals who tested negative for ProGRP. The Hoffmann method, a classic method, was applied to establish the RIs [13]. The GAMLSS approach was employed for continuous RI modeling due to its superior curve-fitting capabilities and effectiveness in mitigating edge effects [30]. These screening criteria and statistical methods ensured high data quality and reliable findings.

This study identified a significant difference in serum NSE levels between males and females. Similar findings have been reported in previous studies [31,32]. However, some have suggested there are no significant sex-related differences in NSE levels [12,33]. Notably, unlike previous studies, we observed a significant difference in NSE levels between females above and below the age of 50 [12,32,33]. This trend, following an S-shaped pattern, was evident in the age-related continuous RI model (Figure 3). While less impactful than age, seasonal effects on NSE levels were still observed (Figure 4). Discrepancies in the influence of sex and age on NSE levels across studies could be attributed to sample size, sampling error, demographic environmental factors, or differences in the choice of detection method(s).

We hypothesize that the age-related variation in female NSE levels may be linked to changes in estrogen levels before and after menopause. Estrogen’s role in regulating mitochondrial function and reducing oxidative stress in neuronal cells is well-documented, and its clinical use in neurological disorders like Alzheimer’s disease is noted [34,35,36]. Therefore, we speculate that NSE, as a biomarker of neuronal cell metabolism and damage, tends to rise as estrogen decreases, particularly during the menopausal transition. Further supporting this hypothesis, NSE levels may not increase in post-menopausal women undergoing hormone replacement therapy with estrogen. This warrants further investigation to assess the validity of our hypothesis and explore the protective role of HRT in maintaining stable NSE levels. This finding suggests that menopause, beyond its hormonal shifts, may influence other biomarkers. Such factors should be carefully considered when establishing IRs for biomarkers in post-menopausal women.

The seasonal fluctuations in NSE levels present a challenge for providing a comprehensive mechanistic explanation. However, parallels with other biomarkers that exhibit seasonal variation—such as thyroid hormones and vitamin D—suggest that environmental factors like temperature and sunlight could play a role [37,38]. Serotonin (5-hydroxytryptamine) levels, which have been shown to fluctuate seasonally with higher levels in fall and winter compared to spring and summer in healthy individuals [39], also show a negative correlation with NSE levels in certain populations [40]. This relationship may point to a broader connection between the seasonal variation in NSE and serotonin activity. The underlying mechanisms of both age and seasonal effects on NSE require further investigation.

In addition to SCLC and the previously discussed neurological diseases, several chronic diseases and lifestyle factors may also influence NSE levels. For example, chronic obstructive pulmonary disease (COPD) has been associated with elevated NSE levels due to hypoxia-induced neuronal damage [41]. Similarly, diabetes can lead to increased NSE levels, particularly in cases where neurological complications such as diabetic neuropathy arise [42]. Moreover, smoking has been identified as a factor influencing NSE levels, potentially due to its role in inducing oxidative stress and neuronal injury [43]. Given these associations, it is essential to account for such covariates when using RIs for health assessments to ensure appropriate interpretation.

Although partitioned RIs are currently utilized in clinical laboratories, they have a limitation in that they cannot accurately reflect the dynamic variations in biochemical analytes influenced by age and the time of sampling [37,44]. This can potentially impact test result interpretations and clinical decisions, especially for individuals near the partition boundaries. While both continuous and partitioned RI upper limits represent the 95th percentile, continuous RIs adjust dynamically with age and/or month, whereas partitioned RIs are most accurate near the center of the partition. In such cases, the upper limit for NSE in partitioned RIs jumps significantly from 14.47 µg/L for 49-year-old females to 17.25 µg/L for 50-year-olds, whereas a continuous age-related model provides a more gradual increase from 15.42 µg/L to 15.63 µg/L, which is more consistent with the expected physiological variations (Figure 3). This discrepancy underscores the need for RI models that reflect the continuous nature of these changes.

Several promising approaches, such as improved non-parametric methods, fractional polynomial regression, the use of GAMs, and the GAMLSS approach, can potentially be utilized for establishing continuous IRs [30,45]. After a comparative evaluation, we selected the GAMLSS approach to model continuous RIs due to its superior performance. GAMLSS were specifically developed to overcome some of the limitations associated with generalized linear models and generalized additive models, and the superior curve-fitting capabilities of such models make the GAMLSS approach a dependable choice for modeling continuous RIs [30,46]. The edge effect is often attributed to insufficient data points for establishing accurate RIs. This scarcity can result in variability due to minor fluctuations in the values observed. To address this challenge, the GAMLSS mode has emerged as an effective statistical approach. GAMLSS allows for the modeling of not just the mean but also the scale and shape of the data distribution, providing a more nuanced understanding and thereby minimizing the edge effect. This model’s flexibility and robustness make it particularly well suited for handling the complexities and variability inherent in the biological data used to establish RIs [30].

Establishing continuous RI models, while more complex than the use of traditional methods, offers significant advantages. Continuous RIs provide a smoother, more accurate representation of individual conditions by avoiding the abrupt changes inherent in partitioned methods, reducing the likelihood of false positive or negative results. As RIs evolve to become more responsive and personalized, they enhance clinical diagnoses’ precision and treatment customization.

Despite their potential, continuous RI models are rarely used in clinical laboratories due to challenges like methodological complexity, standardization difficulties, and technological limitations. However, ongoing research and advancements in laboratory instruments and information systems are paving the way for their adoption. One of the most promising approaches is the integration of continuous RI models directly into the software systems of laboratory instruments. Embedding RI algorithms within analytical equipment allows laboratories to automate the adjustment of RIs based on the patient’s age, sex, and other relevant covariates, also providing the opportunity to flag abnormal results. Modern laboratory information systems and middleware are increasingly being designed to handle complex data analyses. Embedding these models into these systems to enable real-time RI calculations is also a viable solution for the future.

This study also has limitations. Being a real-world study, its reliability and validity are inferior to clinical studies. However, we adopted stringent inclusion and exclusion criteria, as well as robust statistical methods, to ensure that the study had a higher generalizability whilst improving the reliability of the study results. Additionally, edge effects might occur in age-related RI models due to a lack of sufficient data points for individuals aged 70 and above, potentially skewing the representation of this demographic. To address this issue, we employed the GAMLSS approach to minimize edge effects. Future studies should prioritize collecting larger sample sizes for older populations to gain a better understanding of age-related trends in NSE.

## 5. Conclusions

This is the first report of continuous NSE RIs. In this pioneering study, we developed continuous RI models incorporating multiple continuous covariates, highlighting that NSE levels vary with age and season in females. Compared to traditional static RIs, continuous models make RIs more responsive, potentially reducing false positives and negatives, thus enhancing the precision and individualization of health assessments.

## Figures and Tables

**Table 1:** Grouping details based on age and gender.

Age	Gender			
	Male		Female	
	Group	n	Group	n
19–29	1	236	7	269
30–39	2	690	8	583
40–49	3	602	9	436
50–59	4	445	10	276
60–69	5	198	11	187
≥70	6	72	12	63
Total	—	2243	—	1814

**Table 2:** Multiple comparisons among different age groups in both genders (post-log transformation values are shown).

Age Group	Male		Female	
	Meandiff	*p*	Meandiff	*p*
(19–29) vs. (30–39)	−0.009	0.983	−0.002	1
(19–29) vs. (40–49)	−0.023	0.562	0.018	0.787
(19–29) vs. (50–59)	−0.04	0.065	0.163	0
(19–29) vs. (60–69)	−0.036	0.306	0.176	0
(19–29) vs. (>70)	−0.016	0.987	0.158	0
(30–39) vs. (40–49)	−0.013	0.759	0.02	0.477
(30–39) vs. (50–59)	−0.03	0.058	0.165	0
(30–39) vs. (60–69)	−0.026	0.451	0.178	0
(30–39) vs. (>70)	−0.006	1	0.16	0
(40–49) vs. (50–59)	−0.017	0.652	0.145	0
(40–49) vs. (60–69)	−0.013	0.952	0.158	0
(40–49) vs. (>70)	0.007	1	0.14	0
(50–59) vs. (60–69)	0.004	1	0.013	0.968
(50–59) vs. (>70)	0.024	0.899	−0.005	1
(60–69) vs. (>70)	0.02	0.967	−0.018	0.978

## Data Availability

The data presented in this study are only available from the corresponding author upon request due to privacy reasons.

## Supplementary Materials

The following supporting information can be downloaded at: https://www.mdpi.com/article/10.3390/diagnostics14192226/s1, Figure S1: Residual Q-Q plots and worm plots for verifying normality of residuals in GAMLSS models; Table S1: The multiple comparison results of NSE values among different month groups with statistical difference.

## Abbreviations

SCLC, small cell lung cancer; NSE, neuron-specific enolase; RIs, reference intervals; ProGRP, pro-gastrin-releasing peptide; GAMLSS, generalized additive models for location, scale and shape; GAM, generalized additive model.
