# Supplementary material for: Establishing Neuron-Specific Enolase Reference Intervals: A Comparative Analysis of Partitioned Approach- and Gender-Based Continuous Age- and Season-Related Models
Source: Diagnostics (Basel). 2024 Oct 5;14(19):2226. doi: 10.3390/diagnostics14192226 (PMC11475130; doi:10.3390/diagnostics14192226)
Supplement: Supplementary file 1 [file diagnostics-14-02226-s001.zip › Supplementary files.pdf]

Figure S1. Residual Q-Q plots for gender-based continuous age- and season-integrated RI Models

Table S1. The multiple comparison results of NSE values among different month groups with statistical difference. (After log transformation)

Male			Female		
Month group	Meandiff	*P*	Month group	Meandiff	*P*
3 vs 11	0.784	0.01	3 vs 1	1.382	0.007
4 vs 11	0.923	0	3 vs 10	1.859	0
5 vs 11	1.047	0.006	5 vs 10	1.236	0.033
6 vs 11	0.789	0.007	3 vs 11	1.831	0
7 vs 11	0.879	0	4 vs 11	1.252	0.008
8 vs 11	0.86	0	5 vs 11	1.208	0.002
4 vs 12	0.766	0.015	3 vs 12	1.435	0.001
7 vs 12	0.722	0.014	6 vs 3	-1.24	0.006
			7 vs 3	-1.456	0
			8 vs 3	-1.53	0
			9 vs 3	-1.984	0
			9 vs 4	-1.405	0.002
			9 vs 5	-1.361	0
			9 vs 6	-0.744	0.02
