# Supplementary figures and images for: Establishing Neuron-Specific Enolase Reference Intervals: A Comparative Analysis of Partitioned Approach- and Gender-Based Continuous Age- and Season-Related Models
Source: Diagnostics (Basel). 2024 Oct 5;14(19):2226. doi: 10.3390/diagnostics14192226 (PMC11475130; doi:10.3390/diagnostics14192226)

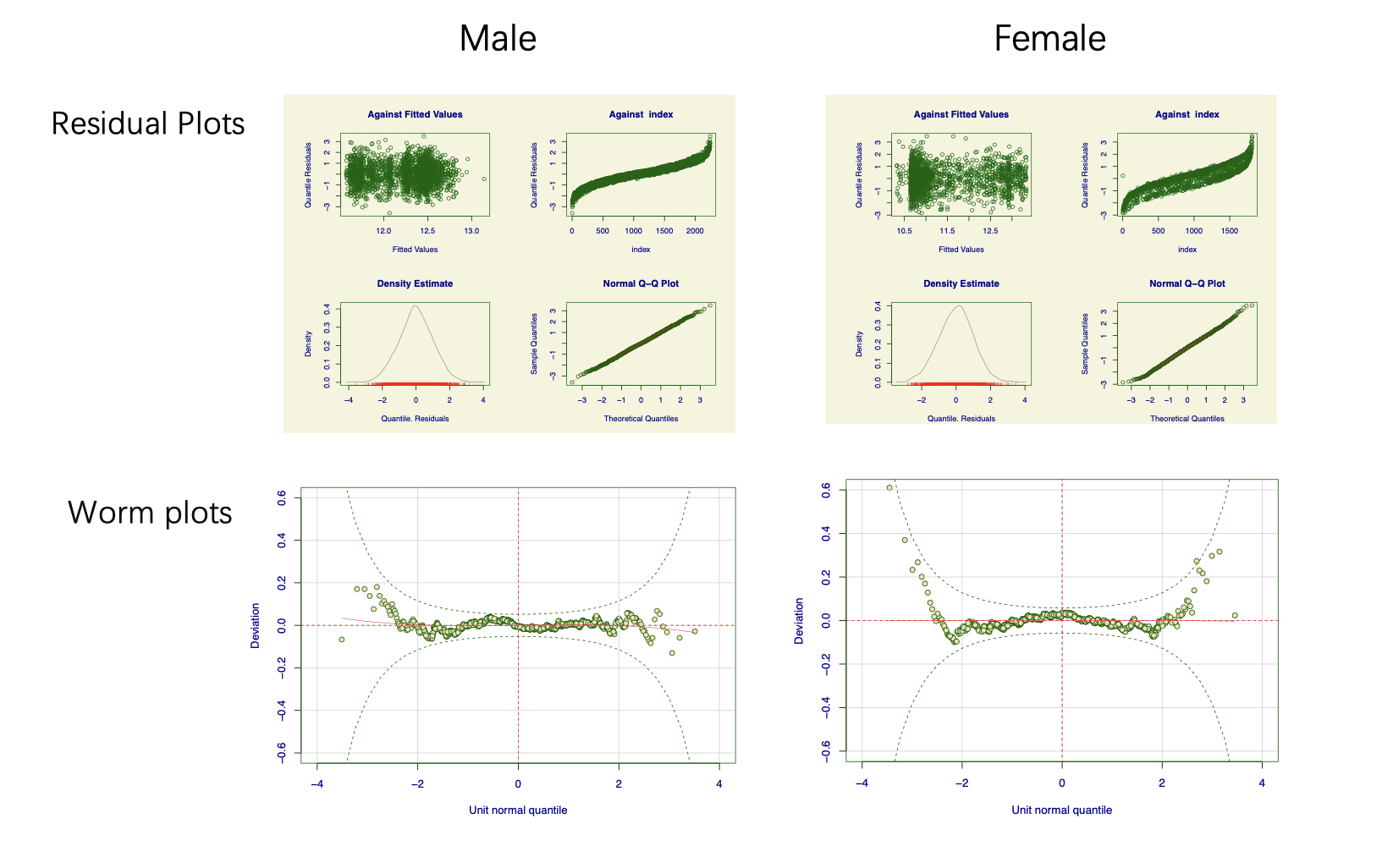

Supplement: Supplementary file 1 [file diagnostics-14-02226-s001.zip › Figure S1.jpg]
